# Allometric co‐variation of xylem and stomata across diverse woody seedlings

**DOI:** 10.1111/pce.13826

**Published:** 2020-07-14

**Authors:** Mengying Zhong, Bruno E. L. Cerabolini, Pilar Castro‐Díez, Jean‐Philippe Puyravaud, Johannes H. C. Cornelissen

**Affiliations:** ^1^ Systems Ecology, Department of Ecological Science Vrije Universiteit Amsterdam Amsterdam The Netherlands; ^2^ Grassland Science Department, College of Grassland Science and Technology China Agricultural University Beijing China; ^3^ Department of Biotechnology and Life Sciences University of Insubria Varese Italy; ^4^ Departamento de Ciencias de la Vida, Facultad de Ciencias Universidad de Alcalá, Carretera Madrid‐Barcelona Madrid Spain; ^5^ Sigur Nature Trust Masinagudi India

**Keywords:** individual stomatal area, individual vessel area, minor vessel number, stomatal number, total stomatal area, xylem cross‐sectional area

## Abstract

Leaf stomatal density is known to co‐vary with leaf vein density. However, the functional underpinning of this relation, and how it scales to whole‐plant water transport anatomy, is still unresolved. We hypothesized that the balance of water exchange between the vapour phase (in stomata) and liquid phase (in vessels) depends on the consistent scaling between the summed stomatal areas and xylem cross‐sectional areas, both at the whole‐plant and single‐leaf level. This predicted size co‐variation should be driven by the co‐variation of numbers of stomata and terminal vessels. We examined the relationships of stomatal traits and xylem anatomical traits from the entire plant to individual leaves across seedlings of 53 European woody angiosperm species. There was strong and convergent scaling between total stomatal area and stem xylem area per plant and between leaf total stomatal area and midvein xylem area per leaf across all the species, irrespective of variation in leaf habit, growth‐form or relative growth rate. Moreover, strong scaling was found between stomatal number and terminal vessel number, whereas not in their respective average areas. Our findings have broad implications for integrating xylem architecture and stomatal distribution and deepen our understanding of the design rules of plants' water transport network.

## Abstract 2

We addressed the hypothesis that the balance of water exchange between the liquid and vapour phase should depend on the convergent scaling between the summed stomatal areas and summed xylem cross‐sectional areas, both at the whole‐plant and single‐leaf level, across diverse woody seedlings of different ecological groups. The attempt has broad implications for integrating xylem architecture and stomatal distribution and for our understanding of whole‐plant water management, also in relation to the West–Brown–Enquist model and the pipe model of vascular hydraulic architecture.

## 1 INTRODUCTION

The xylem system of vascular plants generally features a ‘tip‐to‐base’ widening with the maximal number of the narrowest conduits in the terminal parts; the size of these terminal conduits should not vary with plant size (or leaf size) (Lechthaler, Colangeli, Gazzabin, & Anfodillo, 2019; Rosell & Olson, 2019; West, Brown, & Enquist, 1999). With this hierarchical and basipetally widening xylem architecture, the energy cost of long‐distance water transport is minimized (Anfodillo, Carraro, Carrer, Fior, & Rossi, 2006; Shinozaki, Yoda, Hozumi, & Kira, 1964; West et al., 1999). Under the negative pressure created by stomatal transpiration, water ascends from the soil, progressively through stem and leaf xylem vessels, all the way up to the terminal stomata. Co‐variation of stomatal and xylem traits in leaves is required to maintain a balance in water exchange between the liquid (water delivery) and the vapour (water loss) phase (Brodribb, McAdam, & Carins Murphy, 2017; Carins Murphy, Jordan, & Brodribb, 2014; Zhang, Carins Murphy, Cardoso, Jordan, & Brodribb, 2018).

There is mounting evidence that vein density is proportional to stomatal density in leaves, and this pattern is applicable to diverse plants within and across species (Brodribb et al., 2017; Carins Murphy, Jordan, & Brodribb, 2016; Fiorin, Brodribb, & Anfodillo, 2016). However, the causality of this relationship is difficult to interpret for three reasons. Firstly, leaf vein traits have been proposed to be proxies for leaf xylem properties (Blonder, Violle, Bentley, & Enquist, 2011; Sack et al., 2012). Veins consist of more than xylem (e.g. they also host phloem), so simply considering vein density will ignore xylem vessel number and vessel lumen diameter, which have been deemed the predictors of conductive path length and leaf area respectively (Echeverría, Anfodillo, Soriano, Rosell, & Olson, 2019; Rosell & Olson, 2019). We are not aware of any studies linking stomatal traits to xylem traits per se (i.e. size co‐variation of stomata and xylem vessels) (but see Meinzer and Grantz (1990) about xylem‐stomatal conductance relationships) within and across species. Secondly, stomatal and vein densities reflect leaf water relations in terms of a leaf plane, while it is a system of conduits within a three‐dimensional system, clearly finely tuned by natural selection in a way that directs water nearly optimally given carbon costs, conductance, and embolism resistance (Enquist, 2002; West et al., 1999). Thirdly, these vein‐stomatal density studies (Brodribb et al., 2017; Carins Murphy et al., 2014; Sack, Dietrich, Streeter, Sanchez‐Gomez, & Holbrook, 2008) use the water balance of single leaves to implicate the whole‐plant water balance. This approach might be an oversimplification for understanding the entire liquid phase and the vapour phase relation, even though leaf area has been proved to predict photosynthetic productivity precisely, from the single‐leaf, to the branch, to the whole‐tree, to the forest level (Li et al., 2020).

We address this knowledge gap with a laboratory growth experiment that enabled us to obtain xylem and stomatal traits both at leaf and whole‐plant level. We grew seedlings of 53 diverse woody species from cool‐temperate and Mediterranean Europe in a standard growing environment (Cornelissen et al., 1996; Zhong et al., 2019).

We examined relations between xylem dimensions (Zhong et al., 2019) and stomatal dimensions of these seedlings both at whole‐plant level and at leaf level. Specifically, this study presents, for the first time, the allometric scaling relationships at two scales: (a) between stem xylem cross‐sectional area (as well as stem xylem conductance area) and total stomatal area at the whole‐plant level, and (b) between leaf mid‐vein xylem area and leaf total stomatal area at single‐leaf level. We hereby introduce uniformity in the analyzed pairwise traits as they are expressed in the same physical units, which helps to represent more directly the selection effect on the water flux and enlightens our understanding of the whole‐plant water balance.

**FIGURE 1:**
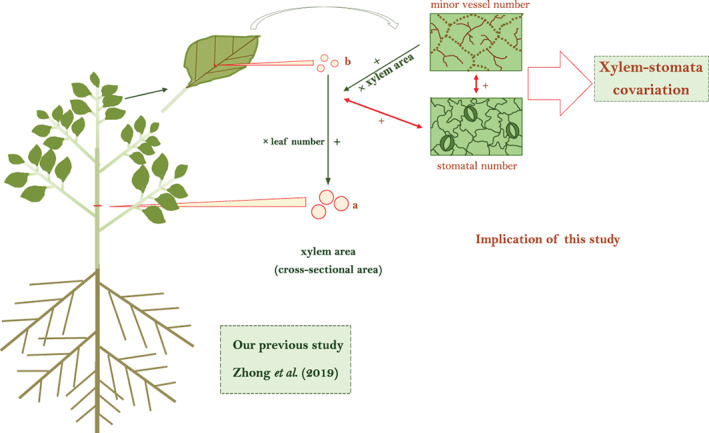
Conceptual framework of this research concerning allometric relations of plant hydraulics across woody species [Colour figure can be viewed at wileyonlinelibrary.com]

Furthermore, as was proposed by the West–Brown–Enquist model, the terminal vessels should be ‘invariant’ with plant size (or leaf size) along with plant growth for a given individual (Roddy et al., 2020; Simonin & Roddy, 2018; West et al., 1999). We tested the relations between terminal vessel traits (i.e. minor vessel number and individual minor vessel area) and plant size (represented by stem xylem area and/or total leaf area per plant) across these 53 species, which were grown in a similar environment, in order to understand whether the terminal vessels ‘depended’ on plant size across diverse species. Additionally, the relations between stomatal traits (i.e. stomatal number and individual stomatal area) and leaf size (represented by midvein xylem area and/or average leaf area) were tested empirically, in order to link with the large body of studies on the allocation mechanism of leaf surface to stomata (Boer et al., 2016; Franks & Farquhar, 2007; Parlange & Waggoner, 1970). Additionally, as stomatal allocation at the leaf surface tends to simultaneously minimize water loss (e.g. water exchange from the minor vessels to stomata) while maximizing gas exchange to maintain a constant photosynthetic productivity per unit leaf area (Boer et al., 2016), we expect that the number of stomata should scale linearly with the number of minor vessels (Figure 1).

Natural selection acts on heritable variation between individuals within the same species. Individuals with vessels that do not widen with height growth, or widen little, will experience continual declines in leaf‐specific conductance with height growth and therefore declining growth and reproductive output per unit leaf area. Individuals with vessels that widen very markedly would have conduits of low resistance, in contrast to the high‐resistance variants with conduits that are ‘too narrow’, but they would have their own set of disadvantages. For example, for a given leaf area and transpirational demand, the wider conduits cost more for the same service provided. Each unit of carbon invested in excessively wide vessels is a unit that is not invested in further growth or reproduction, and so these variants should be at a selective disadvantage (Banavar, Cooke, Rinaldo, & Maritan, 2014). Moreover, wider vessels are more vulnerable to gas embolisms that obstruct conductance, both from freezing ((Sevanto, Holbrook, & Ball, 2012; Zanne et al., 2014) and likely drought as well (Cai & Tyree, 2010; Jacobsen, Pratt, Venturas, & Hacke, 2019; Liu et al., 2019). As a result, plants with vessels that are ‘too wide’ would also be at a selective disadvantage (Zhong et al. (2019)). The variants that should have the largest amounts of surplus carbon to devote to growth and reproduction are those in the intermediate zone, in which conduits widen just enough that conductance remains constant per unit leaf area, but not so much as to incur excessive carbon costs and embolism vulnerability.

In our previous study, based on the same woody seedling populations, we found that, at the whole‐plant level, the stem xylem cross‐sectional area (*X*_stem_) of stem medium (a) closely scales with stem height (*H*) and total leaf area per plant (LA) as *X*_stem_ ∝ *H* ^1.52^ and *X*_stem_ ∝ LA^0.75^ across all the studied species. For individual leaves, vessel diameter (*D*_leaf_) in the medium of leaf midvein (b) closely scales with average leaf area (MLA) as *D*_leaf_ ∝ MLA^0.21^ (Zhong et al. (2019). In this study, we test the poorly understood xylem‐stomata co‐variation from the size perspective. We ask: are there scaling relationships between total stomatal area and xylem cross‐sectional area across species, at the entire plant and at individual‐leaf level? Specifically, we zoom in on the terminal part of water exchange (from minor vessels to stomata), and ask: does the minor vessel number (which scales with leaf area; see Lechthaler et al., 2019; Rosell & Olson, 2019) scale with stomatal number per leaf and per plant across these woody seedlings? The conceptual picture should deepen our understanding of plants' water transport system and have broad implications for integrating xylem architecture and stomatal distribution.

Based on the expectations above, we test the hypothesis that, despite large interspecific differences in leaf‐habit, growth‐form and relative growth rate (RGR), similar scaling should exist between total stomatal area and xylem area across woody seedlings, both at the entire plant and at individual‐leaf level, in a way that ensures the balance between liquid‐ and vapour‐phase water conductance. We also expect that the hypothesized scaling of total stomatal area to xylem area should be driven by the number co‐variation of stomatal and minor vessel elements; we also expect a scaling relation between mean stomatal area and mean minor vessel area as we presumed the distal element size for a given plant to be limited under long‐term nature selection.

## 2 MATERIALS AND METHODS

### 2.1 Seedling growth protocol

Seeds of 53 diverse woody species, belonging to different growth‐forms (19 trees, 22 shrubs, 6 sub‐shrubs and 6 climbers or scramblers) and leaf habits (34 deciduous and 19 evergreens), were collected from cool‐temperate and Mediterranean Europe (Table S1). These species are a sub‐set of those used by Cornelissen et al. (1996), and the seedlings sampled for this anatomical study were sub‐populations of those grown in that growth rate focused study, which was conducted in standard environmental conditions at the Unit of Comparative Plant Ecology, Sheffield University. In brief, throughout 1994 and 1995, all seeds were first germinated and then transplanted into experimental pots that were filled with quarried, prewashed silica sand. An environmental condition of 14 hr 20–22°C:10 hr 15–17°C light:dark was provided, with 135 ± 10 μmol m^−2^ s^−1^ of photosynthetically active radiation [classified as partial shade, see Hendry and Grime (1993)].

The population of each species was evenly divided into two halves for initial and final harvest. After the seedlings opened the first true leaf or leaf pair (i.e. at standardized ontogenetic stage), we harvested the first half population and determined the total plant dry weight. The second half of the population was cultivated for another 21 days within the same standard environment with 0.25 ml per sand volume full‐strength Rorison nutrient solution (N, P and K at 56, 31 and 78 mg L^−1^, respectively, plus Ca, Mg, Fe and trace elements) and sufficient deionized water on alternate days. The seedlings were then harvested, dry weighed and further treated for anatomy analysis [for details see Cornelissen et al. (1996)].

### 2.2 RGR and leaf area

Mean RGR was derived as: RGR = (log_e_ *W*_2_ *−* log_e_ *W*_1_)/(*t*_2_ − *t*_1_), where *W*_1_ and *W*_2_ was the plant dry weight at the first (*t*_1_) and second (*t*_2_) harvest, respectively. At the final harvest, plant total leaf area was measured after saturation in wet tissue paper at 5°C overnight, with a Delta‐T Area Meter (Burwell, Cambridge, UK) for most species, whereas a 1‐mm paper grid was used to calculate leaf area visually for some species with tiny leaves. Average leaf area was calculated as the ratio of total leaf area to leaf number per individual. For each species, 8–30 individuals were used for quantifying the above parameters [for details see Cornelissen et al. (1996), Cornelissen et al. (2003)].

### 2.3 Xylem traits

At the final harvest, three to four seedlings per species were chosen randomly for xylem traits measurements. For each individual, one fully expanded leaf as well as the stem was pickled, and the middle part of each leaf (including the middle of the midvein) as well as the middle of each stem were cut transversely. The materials were embedded in 5% agar and progressively dehydrated in 50, 70 and 95% ethanol (2 hr per solution), after which the small blocks of agar were infiltrated for 15 days with resin JB 4 Polysciences (Polysciences Inc., Warington, PA). After polymerization of the resin, 2‐μm thick cross‐sections were obtained with a glass ultra‐microtome, then sections were stained with 5% toluidine blue and permanently mounted onto slides with DPX (dibutyl phthalate in xylene). The cross‐sections of leaves and stems were studied with a light microscope (Zeiss Axioskop; Carl Zeiss, Jena, Germany) on a computer screen with image analysis software (Aequitas IA v. 1.25; Castro‐Díez, Puyravaud, & Cornelissen, 2000; Castro‐Díez, Puyravaud, Cornelissen, & Villar‐Salvador, 1998).

For stems, stem xylem area and stem xylem conductance area (stem xylem area minus cell wall area) were circled and measured. The proportion of cell wall area relative to xylem area in transverse section was measured in three to four microscopic fields per slide using Aequita tools (Castro‐Díez et al., 1998). For leaves, leaf midvein xylem area and minor vessel area were circled in light microscope images and measured, and the minor vessel area was calculated as the average area of the 10 smallest vessels of the cross‐section of leaves, which were defined as the distal conduits. The plant minor vessel number (*N*_vessel_) was theoretically approximated as: *N*_vessel_ = *A*/(*π* × *R*_s_ × *R*_m_), where *A* is stem xylem conductance area, *R*_s_ is the radius of the biggest vessel in stem medium and *R*_m_ is the radius of the minor vessel in leaves. This calculation was based on the pipe model, which states that the sum of all vessel inner diameters at each vein order is equal (Shinozaki et al., 1964). We used the stem (rather than leaf) xylem conductance area to calculate *N*_vessel_, because it is difficult to gain the leaf xylem conductance area in a representative way from entire leaf cross‐sections, especially for species that have big leaves.

### 2.4 Stomatal traits

At the final harvest, one leaf from each of three different seedlings was randomly selected for epidermal prints; the representative leaf section at about one third from the apex and one third from the midvein was examined. We first brushed acetone onto surfaces of these leaves and then pressed an acetate layer onto them firmly for 30 s and waited for them to dry. Subsequently, we peeled off the acetate layer and mounted it onto a slide for stomatal analysis. Stomatal number of each of 10 randomly selected views (0.12 mm^2^ at ×100) was counted and averaged. When prints did not have sufficient large undamaged and clear areas, smaller areas (0.01–0.05 mm^2^ at ×400) were examined. Stomatal density was determined as the summed number of stomata on both upper and lower surface per one‐sided leaf area. The stomatal area was defined as the area of a guard cell pair, that is, double ellipse 2 × *a* × *b* × *π*, where *a* and *b* are the maximum length and maximum width of the guard cells of 10 randomly selected closed stomata, respectively (Cornelissen et al., 2003). Leaf total stomatal area was defined as stomatal density multiplied by average leaf area and average stomatal area. Correspondingly, plant total stomatal area was defined as stomatal density multiplied by plant total leaf area times average stomatal area. Leaf total stomatal number was defined as stomatal density multiplied by average leaf area. Similarly, plant total stomatal number was defined as stomatal density multiplied by plant total leaf area.

### 2.5 Statistics

Bivariate line fitting of pair‐wise traits across species with contrasting life strategy (i.e. different leaf habits, growth forms and RGRs) was conducted with the standardized major axis (SMA) model using the ‘smatr’ package in R (R Development Core Team, 2014). All data were first ln‐transformed before line fitting. Homogeneity among slopes and Y elevations of fitted lines were determined referring to different groups (leaf habits and growth‐forms). Elevation homogeneity, as well as the overall slope homogeneity with 1, were analyzed when these individual slopes of ecological groups were homogeneous. As the absolute values of stomatal area or xylem area should vary due to different measuring methods, we did not compare the elevations of these regression against the one‐to‐one line. The impact of RGR on these scaling relationships was defined by fitting lines of *Y*/*X* to RGR.

## 3 RESULTS

**FIGURE 2:**
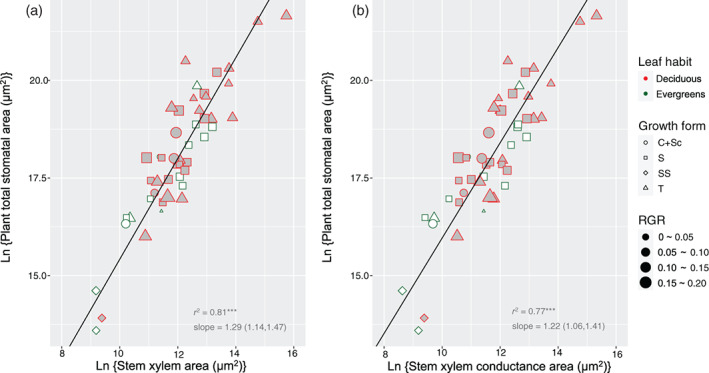
Size co‐variation of stomata and xylem at the whole‐plant level, across seedlings of 53 European woody species varying in leaf habit, growth form and relative growth rate. (a) Convergent scaling of plant total stomatal area and stem xylem transect area. (b) Convergent scaling of plant total stomatal area and stem xylem conductance area. Lines indicate significant scaling relationships. Growth forms: *T*, tree; *S*, shrub; *SS*, sub‐shrub; *C + Sc*, scrambler or climber. Regression coefficients of standardized major axis are documented in Table 1 [Colour figure can be viewed at wileyonlinelibrary.com]

**FIGURE 3:**
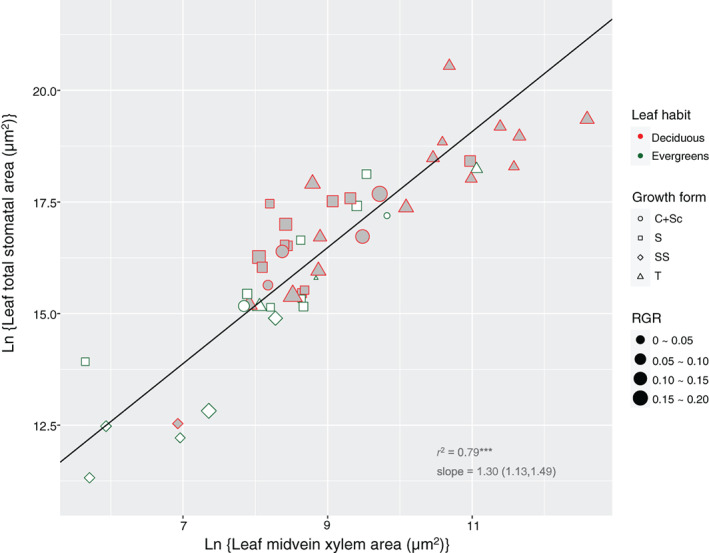
Convergent scaling of leaf total stomatal area and midvein xylem transect area, across seedlings of 53 European woody species varying in leaf habit, growth form and relative growth rate. The line indicates significant scaling relationship. Growth forms: *T*, tree; *S*, shrub; *SS*, sub‐shrub; *C + Sc*, scrambler or climber. Regression coefficients of standardized major axis are documented in Table 1 [Colour figure can be viewed at wileyonlinelibrary.com]

All relations reported below are based on linear SMA regressions on ln‐transformed values. We found strong similarity in scaling relationships across seedlings of woody species between total stomatal area and xylem tissue area from the whole‐plant level to single‐leaf level. Across the 53 species, plant total stomatal area scaled to stem xylem area (slope = 1.29, *r* ^2^ = .81, *p <* .001; Figure 2a), and to stem xylem conductance area (slope = 1.22, *r* ^2^ = .77, *p <* .001; Figure 2b); leaf total stomatal area scaled to midvein xylem area (slope = 1.30, *r* ^2^ = .79, *p <* .001) (Figure 3). The slopes of the regression lines were substantially and significantly larger than 1 (the 95% confidence intervals did not bracket zero; Table 1).

**TABLE 1:** Ln–Ln scaling relationships were analyzed with SMA regression analyses, with additional reference to the contributions of different growth forms, leaf habits and RGRs to these relationships

Model	Intercept (95% CI)	Slope (95% CI)	*r* ^2^ (*p*)	Slope. test = 1, *r* (*p*)	*Y* ~ *X*+ leaf habit (*p*)		*Y* ~ *X* + growth form (*p*)		*Y*/*X* – RGR (*p*)	Figures
					Slope homogeneity	Elevation homogeneity	Slope homogeneity	Elevation homogeneity		
Plant total stomatal area – stem xylem area	2.52 (0.50, 4.55)	1.29 (1.14, 1.47)	.81***	.51***	Ns	Ns	Ns	Ns	Ns	Figure 2a
Plant total stomatal area – stem xylem conductance area	3.76 (1.70,5.82)	1.22 (1.06, 1.41)	.77***	.39**	Ns	Ns	Ns	Ns	Ns	Figure 2b
Leaf total stomatal area – midvein xylem area	4.78 (3.16, 6.39)	1.30 (1.13, 1.49)	.79***	.50***	Ns	Ns	Ns	Ns	Ns	Figure 3
Leaf total stomatal number – midvein xylem area	−1.48 (−3.09, 0.13)	1.32 (1.16, 1.51)	.79***	.53***	Ns	Ns	Ns	Ns	Ns	Figure 4a
Leaf total stomatal number – average leaf area	5.38 (5.02, 5.74)	0.97 (0.91, 1.04)	.94***	−.12, Ns	Ns	Ns	**	—	Ns	
Stomatal cell area – midvein xylem area	8.08 (7.47, 8.68)	−0.22 (−0.30, 0.17)	.01, ns	—	—	—	—	—	—	Figure 4b
Stomatal cell area – average leaf area	6.93 (6.66, 7.20)	−0.16 (−0.22,‐0.13)	.006, ns	—	—	—	—	–	—	
Plant minor vessel number – stem xylem area	−2.73 (−3.86, −1.61)	0.88 (0.79, 0.98)	.87***	—	Ns	**	Ns	**	**	Figure 4c
Plant minor vessel number – total leaf area	3.17 (2.24, 4.10)	0.69 (0.57, 0.83)	.62***	—	Ns	**	Ns	Ns	**	
Minor vessel area – stem xylem area	4.91 (4.08, 5.74)	−0.23 (−0.31, −0.17)	.003, ns	—	—	—	—	—	—	Figure 4d
Minor vessel area – total leaf area	3.46 (3.04, 3.88)	−0.19 (−0.25, −0.14)	.01, ns	—	—	–	—	—	—	
Plant total stomatal number – plant minor vessel number	0.19 (−2.10, 2.47)	1.51 (1.25, 1.82)	.62***	—	Ns	Ns	Ns	—	Ns	Figure 5a
Stomatal cell area – minor vessel area	3.48 (2.71, 4.24)	1.22 (0.91, 1.62)	.05, ns	—	—	—	—	—	—	Figure 5b

*Note: Y*‐intercept and slopes as well as slope homogeneity with 1 are reported for pairwise relationships with significant results. 95% confidence intervals (CI) are in parentheses. Growth‐form: *T*, tree; *S*, shrub; *SS*, sub‐shrub; *C + Sc*, scrambler or climber; Leaf‐habit: *D*, deciduous; *E*, evergreen; *Y*/*X – RGR*, SMA regression of *Y*/*X* (ratio of *Y* values to *X* values) and RGR.

Abbreviations: Ns, not significant; RGRs, relative growth rates; SMA, standardized major axis.

***p < .001; **p < .05.

**FIGURE 4:**
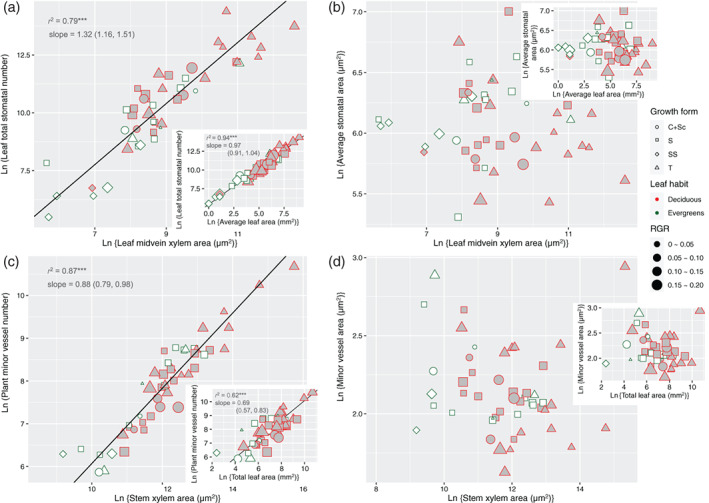
(a) Co‐variation of leaf total stomatal number and midvein xylem area (or average leaf area, insert). (b) Relationship between average stomatal area and midvein xylem area (or average leaf area, insert). (c) Co‐variation of plant total stomatal number and stem xylem area (or total leaf area, insert). (d) Relationship between minor vessel area and stem xylem area (or total leaf area, insert). Lines indicate significant scaling relationships. Growth forms: *T*, tree; *S*, shrub; *SS*, sub‐shrub; *C + Sc*, scrambler or climber. Regression coefficients of standardized major axis are documented in Table 1 [Colour figure can be viewed at wileyonlinelibrary.com]

Moreover, leaf total stomatal number had a strong scaling relation with leaf midvein xylem area (slope = 1.32, *r* ^2^ = .79, *p <*0.001) as well as average leaf area (slope = .97, *r* ^2^ = .97, *p <* .001) (Figure 4a). For the former, the slope of the regression line was significantly larger than 1 (the 95% confidence intervals did not bracket zero), while slope of the latter was convergent to 1 (Table 1). In contrast, average stomatal area was independent of leaf midvein xylem area (*r* ^2^ = .01, *p* > .05) or average leaf area (*r* ^2^ *<* .01, *p* > .05) (Figure 4b; Table 1). Similarly, plant minor vessel number scaled with stem xylem area (slope = 0.88, *r* ^2^ = .88, *p <* .001) and total leaf area (slope = 0.69, *r* ^2^ = .62, *p <* .001; Figure 4c) (Table 1). Average minor vessel area was independent of stem xylem area (*r* ^2^ *<* .01, *p* > .05) or total leaf area (*r* ^2^ *<* .01, *p* > .05) (Figure 4d; Table 1).

**FIGURE 5:**
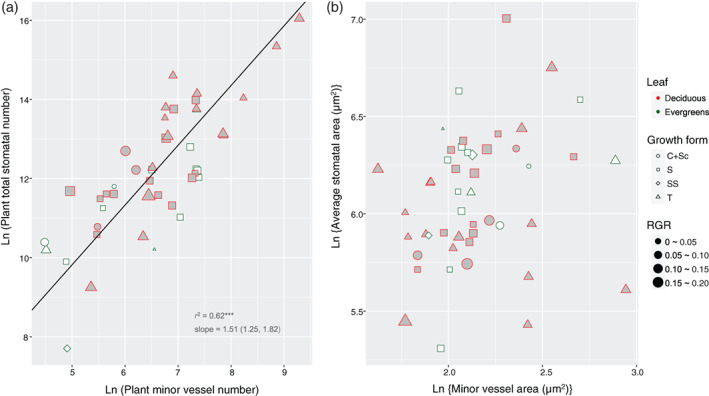
(a) Co‐variation of plant total stomatal number and plant minor vessel number. (b) Relation between average stomatal area and average minor vessel area. The line indicates significant scaling relationship. Growth forms: *T*, tree; *S*, shrub; *SS*, sub‐shrub; *C + Sc*, scrambler or climber. Regression coefficients of standardized major axis are documented in Table 1 [Colour figure can be viewed at wileyonlinelibrary.com]

Furthermore, plant total stomatal number strongly scaled with plant minor vessel number (slope = 1.51, *r* ^2^ = .62, *p <* .001; Figure 5a); while the average area of stomata and minor vessels did not show any relation with each other (*r* ^2^ *=* .05, *p* > .05; Figure 5b) (Table 1).

## 4 DISCUSSION

Woody seedlings are a convenient life stage to acquire water conductance parameters at the whole‐plant level because of their size advantage, even though patterns of seedlings may could not completely reflect the water relation in adult woody plants. Nevertheless, how woody seedlings regulate water relations, in terms of the xylem‐stomatal co‐variation, is important for their survival and growth into adulthood. Based on an anatomical analysis across ontogenetically comparable seedlings of 53 diverse woody species (Table S1), we have presented here key new findings on xylem‐stomata co‐ordination from a previously neglected aspect: we started from the water balance between the liquid (water delivery) and vapour (water loss) phase at the whole‐plant level by scaling plant total stomatal area to stem xylem (conductance) cross‐sectional area (Figure 2; Table 1). This area‐scaling pattern was driven by the co‐variation of stomata numbers and minor vessel numbers per plant (Figure 5a; Table 1). We then zoomed in on the water exchange in individual leaves by showing the co‐ordination of leaf total stomatal area and midvein xylem area (Figure 3; Table 1). We also found that plant size (or leaf size) scales with stomatal (or minor vessel) number, while it does not scale with individual stomatal (or minor vessel) area (Figure 4; Table 1), which has an important implication for our understanding of the design of xylem structure and stomatal distribution.

### 4.1 Convergent size co‐ordination of stomata and xylem from whole‐plant level to single‐leaf level

The convergent size co‐ordination of stomata and xylem in the case of entire plants and individual leaves implies that individual leaves have a tight control over the whole‐plant water conductance (Figures 2 and 3; Table 1). Previously we showed, based on the same seedling populations, that the xylem vessels widen basipetally from the tip to the base for both single leaf (midvein) and the whole plant (stem) and that the remarkably tight co‐variation in vessel diameter between different organs (especially between leaf and stem) (Zhong et al., 2019). When we now combine all three findings, we can conclude that natural selection has led to rather tight regulation of water‐related architecture featuring similar size‐driven variation across seedlings of diverse woody species, both for single leaves and entire plant individuals. Their xylem vessels widen basipetally from the tip to the base, from leaves to the entire individuals, in a way that maintains a constant leaf‐specific conductance (Sterck & Zweifel, 2016; Zhong et al., 2019) and a constant xylem‐stomatal size scaling. Using hydraulic properties of single leaves to predict the entire plant water transport is an alternative choice, as numerous studies have done (Brodribb et al., 2017; Carins Murphy et al., 2014; Meinzer, 2002), especially when the conductance‐related parameters of entire plants are difficult to acquire, for example in adult trees. Specifically, knowing leaf size (i.e. leaf area) is of the utmost importance, not only to predict photosynthetic productivity precisely (Li et al., 2020), but also to understand plant water transport (Echeverría et al., 2019), from the single leaf, to the branch, to the whole‐tree, and even to the forest level.

The slopes of the ln‐scaling regression lines between stomatal and xylem traits are notably larger than the slope of 1 (Figures 2 and 3; Table 1), which means that stomata do not scale linearly to xylem but exponentially. In actual fact, it should be the stem xylem conductance area, rather than stem xylem area, that scales with the total stomatal area, whereas we gave the pattern for both in order to enable comparison with a previous study which used the same seedling population (Zhong et al., 2019). In that study, the total leaf area (LA) scaled with stem xylem area (*X*_stem_) at mid stem height as LA ∝ *X*_stem_^1.25^ (Zhong et al., 2019). Together with isometric scaling of leaf area and total stomatal area, we could elicit that total stomatal area should scale with *X*_stem_ with an exponent approximating 1.25. Our finding in the current paper (exponents 1.29 and 1.30 for entire plants and individual leaves, respectively) is in line with this theoretical prediction. When considering the total water path length (e.g. by sampling the anatomical cross‐section at the stem base), our results are in line with our prediction that there should be isometric scaling both between total leaf area and xylem conductance area and between total stomatal conductance area and xylem conductance area (Echeverría et al., 2019; Fiorin et al., 2016; Lechthaler et al., 2019; Meinzer & Grantz, 1990). Further studies are needed to integrate the relations between leaf area, stomatal area and xylem conductance area from the perspective of the (three dimensional) water transport system from single‐leaf level to whole‐plant level. Ideally, such studies should be carried out also on adult woody plants and across different biomes.

### 4.2 Number co‐ordination of stomata and minor vessels and its implication

We also found strong co‐variation between terminal xylem vessel number and stomatal number per plant (Figure 5a; Table 1). That is: in order to ensure the balance between liquid‐ and vapour‐phase water conductance, convergent scaling exists between total stomatal area and xylem area, both at the entire plant and at individual‐leaf level; and this area‐scaling pattern was driven by the co‐variation of stomata numbers and minor vessel numbers per plant.

These findings provide empirical support for, as well as a better functional understanding of the xylem structure models and have broad implications for integrating xylem widening (Anfodillo et al., 2006; Olson et al., 2014; Zhong et al., 2019) and stomatal distribution; these linkages are illustrated in Figure 1. Further studies on plant water relations should incorporate the transport mechanism of water from the minor vein xylem vessels to stomata with xylem architecture.

## 5 CONCLUSION

Woody seedlings across ecologically and morphologically wide‐ranging species modulate the balance between the vapour (water loss) and liquid (water delivery) phase, via a convergent allometric co‐variation of xylem area and total stomatal area from entire individuals to individual leaves. Having a sufficient number of stomata relative to the minor vein xylem number is imperative for ensuring the force (generated by evaporation through stomata) of water delivery (through xylem vessels). The whole‐leaf and whole‐plant allometric relationships related to water transport and export in this study deepen our understanding of the vascular structure models and has broad implications for integrating xylem architecture and stomatal distribution across species.

## CONFLICT OF INTEREST

The authors declare no potential conflict of interest.

## AUTHOR CONTRIBUTIONS

J.H.C.C., B.E.L.C., P.C.‐D. and J.‐P.P. carried out the experimental work. J.H.C.C., B.E.L.C., P.C.‐D., J.‐P.P. and M.Y.Z. gathered the data. M.Y.Z. and J.H.C.C. conceived the present study and designed the analyses. M.Y.Z. analyzed the data and wrote the first draft of the manuscript. All authors made important suggestions on at least two drafts of the manuscript.

**Definitions of parameters**
Average leaf area: mean area of single leaves (mm^2^)
Average stomatal area: mean area of a guard cell pair, that is, double ellipse 2*a*b*π, where *a* and *b* are the maximum length and maximum width of the guard cells (μm^2^)
Leaf total stomatal area: the sum of all stomatal areas per leaf (μm^2^)
Minor vessel area: mean area of plant terminal vessels (μm^2^)
Plant minor vessel number: the sum number of all terminal vessels per plant
Plant total stomatal area: the sum of all stomatal areas per plant (μm^2^)
Plant total stomatal number: the sum number of all stomata per plant
Stem xylem area: cross‐sectional area of the entire xylem tissue in stems (μm^2^)
Stem xylem conductance area: cross‐sectional area of the conducting section in stem xylem (μm^2^)
Total leaf area: the sum of all leaf areas per plant (mm^2^)

## Supporting information


**Table S1** Growth‐form, Leaf‐habit and seedling traits of the 53 studied woody species. Growth‐form: *T* tree, *S* shrub, *SS* sub‐shrub, *C + Sc* scrambler or climber; Leaf‐habit: *D* deciduous, *E* evergreen. *RGR* relative growth rate (dataset could be found in Cornelissen, Castro‐Díez, and Hunt (1996)). Majority part of these datasets – *Stem xylem area*, *Leaf (midvein) xylem area*, *Total leaf area* and *Average leaf area* – could be found in (Zhong, Castro‐Díez, Puyravaud, Sterck, & Cornelissen, 2019). The dataset of *average stomatal area* could be found in (Cornelissen et al., 2003). The entire dataset of this research was provided here for easy access.
