# Supplementary material for: Allometric co‐variation of xylem and stomata across diverse woody seedlings
Source: Plant Cell Environ. 2020 Jul 14;43(9):2301–10. doi: 10.1111/pce.13826 (PMC7496827; doi:10.1111/pce.13826)
Supplement: Supplementary file 1 — Table S1 Growth‐form, Leaf‐habit and seedling traits of the 53 studied woody species. Growth‐form: T tree, S shrub, SS sub‐shrub, C + Sc scrambler or climber; Leaf‐habit: D deciduous, E evergreen. RGR relative growth rate (dataset could be found in Cornelissen, Castro‐Díez, and Hunt (1996)). Majority part of these datasets – Stem xylem area, Leaf (midvein) xylem area, Total leaf area and Average leaf area – could be found in (Zhong, Castro‐Díez, Puyravaud, Sterck, & Cornelissen, 2019). The dataset of average stomatal area could be found in (Cornelissen et al., 2003). The entire dataset of this research was provided here for easy access. [file PCE-43-2301-s001.docx]

**Table S1** Growth-form, Leaf-habit and seedling traits of the 53 studied woody species. Growth-form: *T* tree, *S* shrub, *SS* subshrub, *C+Sc* scrambler or climber; Leaf-habit: *D* deciduous, *E* evergreen. *RGR* relative growth rate (dataset could be found in Cornelissen *et al.* (1996)). Majority part of these datasets -*Stem xylem area*, *Leaf (midvein) xylem area*, *Total leaf area* and *Average leaf area*- could be found in (Zhong *et al.*, 2019). The dataset of *average stomatal area* could be found in (Cornelissen *et al.*, 2003). The entire dataset of this research was provided here for easy access.

| Species | Growth form | Leaf habit | RGR | Plant total stomatal area (μm^2^) | Leaf total stomatal area (μm^2^) | Leaf total stomatal number | Average stomatal area (μm^2^) | Stem xylem area (μm^2^) | Xylem conductance area (μm^2^) | Leaf midvein xylem area (μm^2^) | Minor vessel area (μm^2^) | Total leaf area (mm^2^) | Average leaf area (mm^2^) |
| --- | --- | --- | --- | --- | --- | --- | --- | --- | --- | --- | --- | --- | --- |
| *Acer platanoides* | T | D | 0.0755 | 223804929 | 106603260 | 468138 | 228 | 343755 | 162768 | 34838 | 11.26 | 4449 | 2119 |
| *Acer pseudoplatanus* | T | D | 0.0808 | 186377155 | 89310035 | 232903 | 383 | 1075797 | 680388 |  | 11.50 | 3093 | 1482 |
| *Aesculus hippocastanum* | T | D | 0.0935 | 2537142026 | 253714203 | 929899 | 273 | 6874015 | 4529976 | 293560 | 18.97 | 43988 | 4399 |
| *Alnus glutinosa* | T | D | 0.1106 | 23416451 | 8519951 | 13635 | 625 | 188371 | 130840 | 7134 | 10.91 | 407 | 148 |
| *Arbutus unedo* | T | E | 0.0919 | 14208189 | 3830456 | 7225 | 530 | 31345 | 16743 | 3157 | 17.95 | 204 | 55 |
| *Berberis vulgaris* | S | D | 0.0844 | 21345164 | 5157241 | 9532 | 541 | 96960 | 39624 | 5684 | 14.38 | 354 | 85 |
| *Betula pendula* | T | D | 0.1280 | 8928559 | 3989847 | 4665 | 855 | 53181 | 37014 | 2738 | 12.80 | 113 | 50 |
| *Buddleja davidii* | S | D | 0.2000 | 66779051 | 11589874 | 20622 | 562 | 55563 | 38530 | 3137 | 9.06 | 861 | 149 |
| *Buxus sempervirens* | S | E | 0.0579 | 14440719 | 3759371 | 5187 | 725 | 27728 | 12212 | 3684 | 14.88 | 169 | 44 |
| *Calluna vulgaris* | SS | E | 0.0541 |  | 82431 | 192 | 428 | 5206 | 3202 | 301 |  |  | 1 |
| *Castanea sativa* | T | D | 0.0746 | 659912449 | 173246507 | 366410 | 473 | 951942 | 518570 | 114990 | 6.70 | 8352 | 2193 |
| *Cornus sanguinea* | S | D | 0.1028 | 56448859 | 14903255 | 40747 | 366 | 159184 | 100657 | 4650 | 7.21 | 1177 | 311 |
| *Corylus avellana* | S | D | 0.1309 | 597659121 | 99609853 | 196083 | 508 | 626847 | 388287 | 58223 | 7.68 | 11584 | 1931 |
| *Crataegus monogyna* | S | D | 0.1059 | 59703754 | 11651733 | 20795 | 560 | 226117 | 114415 |  | 7.49 | 1306 | 255 |
| *Cytisus scoparius* | S | E | 0.0918 | 41138286 | 5072802 | 25123 | 202 | 174629 | 92112 | 2666 | 7.09 | 1014 | 125 |
| *Empetrum nigrum* | SS | E | 0.0587 | 2220093 | 261786 | 595 | 440 | 9699 | 5593 | 378 | NA | 17 | 2 |
| *Fagus sylvatica* | T | D | 0.0412 | 304853187 | 152426594 | 375785 | 406 | 280394 | 154667 | 39645 | 5.87 | 3389 | 1694 |
| *Frangula alnus* | S | D | 0.1164 | 38324802 | 9223801 | 26465 | 349 | 117499 | 60241 | 3283 | 8.26 | 501 | 121 |
| *Fraxinus excelsior* | S | E | 0.1017 | 147861778 | 36789017 | 90052 | 409 | 536288 | 297372 |  | 7.92 | 3971 | 988 |
| *Hebe x franciscana* | C+Sc | E | 0.1025 | 12333030 | 3866248 | 10173 | 380 | 27081 | 16023 | 2547 | 9.71 | 69 | 22 |
| *Hedera helix* | C+Sc | E | 0.0257 | 68535534 | 29306295 | 56902 | 515 | 86503 | 54324 | 18421 | 11.33 | 442 | 189 |
| *Helianthemum nummularium* | SS | E | 0.1129 |  | 2941751 | 5383 | 546 | 39081 | 15315 | 3940 | 8.39 |  | 17 |
| *Hippophae rhamnoides* | S | D | 0.0744 | 37317548 | 5519993 | 14444 | 382 | 64016 | 39092 | 5895 | 8.43 | 336 | 50 |
| *Ilex aquifolium* | T | E | 0.0137 | 17026164 | 7218047 | 11564 | 624 | 92510 | 92510 | 6906 | 7.17 | 96 | 41 |
| *Juglans regia* | T | D | 0.0643 | 2186734626 | 838486789 | 1765820 | 475 | 2564331 | 2564331 | 43642 | 6.73 | 21178 | 8121 |
| *Laburnum anagyroides* | T | D | 0.0824 | 62620395 | 18136573 | 62059 | 292 | 176117 | 176117 | 7302 | 11.31 | 2016 | 584 |
| *Ligustrum vulgare* | S | E | 0.072 | 92734988 | 16910829 | 30565 | 553 | 237776 | 237776 | 5586 | 8.18 | 608 | 111 |
| *Lonicera periclymenum* | C+Sc | D | 0.0773 | 27182339 | 6170674 | 10937 | 564 | 73158 | 46415 | 3551 | 10.59 | 295 | 67 |
| *Malus sylvestris* | T | D | 0.1087 | 181004476 | 35085012 | 98027 | 358 | 516712 | 516712 | 23994 | 7.81 | 2685 | 520 |
| *Prunus laurocerasus* | S | E | 0.0794 | 156932580 | 74277221 | 97945 | 758 | 302786 | 302786 | 13868 | 7.81 | 1806 | 855 |
| *Prunus lusitanica* | S | E | 0.1001 | 113721032 | 36432187 | 64014 | 569 | 406418 | 406418 | 12132 | 7.93 | 1272 | 407 |
| *Prunus spinosa* | S | D | 0.1416 | 345051252 | 40525534 | 110922 | 365 | 407299 | 251252 | 8692 | 8.43 | 4620 | 543 |
| *Quercus cerris* | T | D | 0.0645 | 794869756 | 215056379 | 592037 | 363 | 212333 | 212333 | 88171 | 6.55 | 4258 | 1152 |
| *Quercus ilex ilex* | T | E | 0.0630 | 419506213 | 83111179 | 184718 | 450 | 316025 | 316025 | 63637 | 8.33 | 2197 | 435 |
| *Quercus petraea* | T | D | 0.0615 | 320599019 | 67059123 | 198294 | 338 | 430118 | 430118 | 59064 | 7.57 | 3610 | 755 |
| *Quercus robur* | T | D | 0.0472 | 446422644 | 87661045 | 244593 | 358 | 939504 | 939504 | 106646 | 5.96 | 3530 | 693 |
| *Rhamnus alaternus* | S | E | 0.054 | 23379549 | 4553052 | 15014 | 303 | 63217 | 27894 | 5654 | 7.44 | 243 | 47 |
| *Rhamnus cathartica* | S | D | 0.0720 | 56354993 | 15417573 | 50842 | 303 | 172466 | 172466 | 4471 | 6.27 | 586 | 160 |
| *Rhododendron ponticum* | S | E | 0.0570 |  | 1111068 | 2457 | 452 | 16204 | 16204 | 284 | 7.78 |  | 10 |
| *Ribes nigrum* | S | D | 0.1761 | 223802657 | 24133735 | 48511 | 497 | 168779 | 168779 | 4537 | 8.48 | 2422 | 261 |
| *Ribes uva-crispa* | S | D | 0.1207 | 48489246 | 10525089 | 17938 | 587 | 207779 | 207779 |  | 7.99 | 992 | 215 |
| *Rosa arvensis* | C+Sc | D | 0.1439 | 65827185 | 13143722 | 40272 | 326 | 142694 | 86488 | 4328 | 6.27 | 1672 | 334 |
| *Rubus fruticosus* | C+Sc | D | 0.1778 | 127053969 | 18354321 | 47090 | 390 | 153453 | 110145 | 13137 | 9.18 | 2335 | 337 |
| *Salix caprea* | T | D | 0.1913 | 24352804 | 4847936 | 20860 | 232 | 114703 | 114703 | 5003 | 5.87 | 560 | 112 |
| *Sambucus nigra* | S | D | 0.1393 | 181928719 | 43208882 | 39266 | 1100 | 408946 | 408946 | 11100 | 10.05 | 2209 | 525 |
| *Solanum dulcamara* | C+Sc | D | 0.2271 |  | 47749004 | 153250 | 312 | 256711 | 172567 | 16659 | 8.16 |  | 599 |
| *Sorbus aucuparia* | T | D | 0.1167 | 36145255 | 10221650 | 19561 | 523 | 80505 | 80505 |  |  | 696 | 197 |
| *Thymus polytrichus* | SS | E | 0.1308 |  | 369414 | 922 | 401 | 40231 | 40231 | 1567 |  |  | 3 |
| *Ulex europaeus* | S | E | 0.0781 | 32604769 | 3818019 | 7178 | 532 | 192394 | 192394 | 5807 | 7.36 | 293 | 34 |
| *Ulmus glabra* | T | D | 0.1200 | 240512411 | 60001258 | 118453 | 507 | 131718 | 131718 | 6578 | 5.08 | 1975 | 493 |
| *Vaccinium myrtillus* | SS | D | 0.0524 | 1102862 | 277584 | 805 | 345 | 11885 | 11885 | 1021 |  | 11 | 3 |
| *Vaccinium vitis-idaea* | SS | E | 0.0492 | 801863 | 201557 | 558 | 361 | 9745 | 9745 | 1054 | 6.65 | 11 | 3 |
| *Viburnum opulus* | S | D | 0.0767 | 66860566 | 38275083 | 62941 | 608 | 93487 | 50483 | 3639 | 9.62 | 721 | 412 |
